# The Depletion of Carbohydrate Metabolic Genes in the Gut Microbiome Contributes to the Transition From Central Obesity to Type 2 Diabetes

**DOI:** 10.3389/fendo.2021.747646

**Published:** 2021-10-22

**Authors:** Ruikai Jia, Min Huang, Lichun Qian, Xiaoye Yan, Qing Lv, Hua Ye, Li Ye, Xin Wu, Weizhi Chen, Ye Chen, Yankai Jia, Yueqing Huang, Huihui Wu

**Affiliations:** ^1^ GENEWIZ Inc., Suzhou, China; ^2^ Department of General Medicine, The Affiliated Suzhou Hospital of Nanjing Medical University, Suzhou, China

**Keywords:** central obesity, type 2 diabetes, transition, biomarker, gut microbiome, metagenomics

## Abstract

Obesity, especially central obesity, is a strong risk factor for developing type 2 diabetes (T2D). However, the mechanism underlying the progression from central obesity to T2D remains unknown. Therefore, we analyzed the gut microbial profiles of central obese individuals with or without T2D from a Chinese population. Here we reported both the microbial compositional and gene functional alterations during the progression from central obesity to T2D. Several opportunistic pathogens were enriched in obese T2D patients. We also characterized thousands of genes involved in sugar and amino acid metabolism whose abundance were significantly depleted in obese T2D group. Moreover, the abundance of those genes was negatively associated with plasma glycemia level and percentage of individuals with impaired plasma glucose status. Therefore, our study indicates that the abundance of those depleted genes can be used as a potential biomarker to identify central obese individuals with high risks of developing T2D.

## Introduction

Obesity and type 2 diabetes (T2D) are metabolic disorders with increasing incidence rates both worldwide (1) and within China (2). The International Diabetes Federation estimated that the number of people with diabetes was 463.0 million in 2019 and is expected to rise to 578.4 and 700.2 million by 2035 and 2045, respectively (1). Obesity, particularly central (intra-abdominal) obesity, is a well-established strong risk factor for T2D (3). Nevertheless, all obese individuals do not develop T2D (4, 5). Currently, the causal factors driving or preventing the transition from obesity to T2D remain unknown. However, numerous factors have been reported to play important roles in developing T2D, including genetic background and personal lifestyle, many of which have also been linked to the gut microbiome (6, 7). Thus, it is worthwhile to identify the distinguishing microbiome markers driving the transition from obesity to T2D. Such markers will contribute to a better understanding of the pathophysiological mechanism of progressing from obesity to T2D.

Increasing evidence has demonstrated the close relationship between the microbiome and various human diseases, especially metabolic-related diseases (8, 9). There is general agreement that the loss of a butyrate producer microbiota is associated with T2D in different cohorts with different ethnicities (3, 9, 10). Furthermore, the loss of those butyrate producers may contribute to low-grade inflammation, dysregulated glucose metabolism, and insulin resistance; thus, it is associated with the progression of T2D (3, 11, 12). Given these common observations, the attempts to identify an association between a single microbe and T2D have yielded inconsistent results (13, 14). The discrepancies may be due to the differences in trial designs and the influence of confounders, such as medical treatment (15). It has been well reported that medical treatment, such as metformin, greatly impacts on the gut microbiome taxonomic composition and ecological diversity (11, 16).

Thus, in this study, to exclude the influences of those confounders, we performed 16S rRNA gene sequencing and metagenomic sequencing analysis of 60 central obesity non-diabetic participants and 183 central obesity patients who were newly diagnosed as T2D and hence medicine treatment-naïve. We aim to identify the potential compositional and/or functional features of the gut microbiota that contribute to the transition from central obesity to T2D.

## Materials and Methods

### Study Cohort and Patient Characteristics

We collected the stool samples of 243 adults for this study. The sample collection was approved by the ethics committee of Suzhou Municipal Hospital. All volunteers signed a written informed consent before sample collection. The volunteers were classified into two groups (183 in the experimental group and 60 in the control group). The experimental group was the abdominal obesity patients who were newly diagnosed with type 2 diabetes mellitus (waist circumference, man ≥90 cm; woman ≥85 cm; fasting plasma glucose level >7 mmol/L) (the criteria of weight for Chinese adults is according to the instructions of the National Health Commission of the People’s Republic of China, http://www.nhc.gov.cn/wjw/yingyang/201308/a233d450fdbc47c5ad4f08b7e394d1e8.shtml), so no treatment medicines had been taken. Abdominal obese individuals who did not have diabetes served as control.

The exclusion criteria for the two groups include (1) antibiotic consumption during the last 2 months, (2) family history of diabetes or other related diseases, (3) major gastrointestinal surgery and any known chronic disorders, (4) pregnancy at the time of sample collection, and (5) hypothyroidism.

### Stool Samples Collection and DNA Extraction

The stool samples were collected using the stool collection kit, Flora Prep, provided by Admera Health Inc. (Suzhou, China). After collection, the samples were stored at 4°C and sent to the laboratory within 1 to 2 days. As instructed, genomic DNA was extracted from the stool samples using a Qiagen DNeasy PowerSoil HTP 96 kit (cat. no. 12955-4). The Qubit dsDNA HS assay kit was used to determine the concentration and quality of the purified DNA.

### 16S rRNA Gene Amplicon Preparation and Sequencing

Purified DNA (20–30 ng) was used to generate amplicons. The V3 and V4 hypervariable regions of prokaryotic 16S rRNA were selected for generating amplicons and following taxonomy analysis. These regions were amplified using forward primers containing the sequence “CCTACGGRRBGCASCAGKVRVGAAT” and reverse primers containing the sequence “GGACTACNVGGGTWTCTAATCC” (patent no. US 9745611B2). Finally, indexed adapters were added to the ends of the 16S rRNA amplicons to generate indexed libraries ready for downstream next-generation sequencing on an Illumina MiSeq platform.

The PCR reactions were performed in triplicates of 25-μl mixtures containing 2.5 μl TransStart Buffer, 2 μl of dNTPs, 1 μl of each primer, and 20 ng template DNA.

The DNA library concentration was determined using the Qubit 3.0 Fluorometer. The library was quantified to 10 nM. The DNA libraries were multiplexed and loaded on an Illumina MiSeq instrument with pair-end 250-bp (PE250) mode according to the instructions of the manufacturer (Illumina, San Diego, CA, USA). Image analysis and base calling were conducted by the control software embedded in the instrument. All the 16S rRNA gene sequencing experiments were conducted by GENEWIZ Inc. (Suzhou, China).

### Metagenomic Sequencing

Metagenomic libraries were constructed according to the protocol of the manufacturer (Vazyme, VAHTS Universal DNA Library Prep Kit for Illumina, cat. ND607-01). For each sample, 200 ng of genomic DNA was randomly fragmented to <500 bp by sonication (Covaris S220). The fragments were treated with End Prep Enzyme Mix for end repairing, 5′ phosphorylation, and dA-tailing in one reaction, followed by a T-A ligation to add adaptors to both ends. Size selection of adaptor-ligated DNA was then performed using VAHTS DNA Clean Beads (Vazyme, N411-01), and fragments of approximately 470 bp (with an approximate insert size of 350 bp) were recovered. Each sample was then amplified for eight cycles using Illumina P5 and P7 indexed primers, with both primers carrying sequences, which can anneal with the flow cell to perform bridge PCR and P7 primer carrying a six-base index allowing for multiplexing. The PCR products were cleaned up using VAHTS DNA Clean Beads, and the fragment size can be visualized using an Agilent 2100 Bioanalyzer (Agilent Technologies, Palo Alto, CA, USA) and quantified by Qubit 3.0 Fluorometer (Invitrogen, Carlsbad, CA, USA).

Then, libraries with different indexes were multiplexed and loaded on an Illumina HiSeq X instrument according to the instructions of the manufacturer’s (Illumina, San Diego, CA, USA). Sequencing was conducted using a paired-end 150-bp (PE150) mode. Image analysis and base calling were conducted by the HiSeq Control Software (HCS) + OLB + GAPipeline-1.6 (Illumina). All metagenomic sequencing experiments were conducted by GENEWIZ Inc. (Suzhou, China).

### 16S rRNA Gene Sequencing Data Processing

Paired-end reads were firstly assembled to filter reads smaller than 200 bp with N base. Then, chimeric reads were removed, and the resulting sequences were used for downstream operational taxonomic unit (OTU) clustering with the VSEARCH program (v1.9.6) (17). Sequences with similarities larger than 97% were classified as the same OTU. The annotations of taxonomic information were achieved from the Ribosomal Database Project classifier algorithm (http://sourceforge.net/projects/rdp-classifier) according to the SILVA_132 database (18).

Based on OUT taxonomic annotation information, alpha diversity indices, such as Shannon and Chao1, and rarefaction curves and rank–abundance graphs reflecting species richness and evenness were calculated by the methods implemented in Quantitative Insights Into Microbial Ecology (QIIME) v1.9.1 (19). Venn diagrams of the overlapping OTUs between two groups were drawn by using the package in R. The principal coordinate analysis (PCoA) and the nonmetric multidimensional scaling (NMDS) analysis are based on the Bray–Curtis distances.

### Metagenomic Sequencing Data Processing

Raw shotgun sequencing reads were trimmed using cutadapt (v1.9.1) (20). Reads with low-quality and N bases and adapter-contaminated were removed. Then, human host contamination reads were removed by mapping the sequencing reads to the human genome (hg38) with BWA (v0.7.12) (21). Finally, the remaining reads were assembled *de novo* with MEGAHIT(v1.13) (22) using different k-mer to obtain separate assemblies in each individual. The best assemble scaffold with the largest N50 was selected for downstream gene prediction analysis.

To identity the composition of microbial communities from metagenomic shotgun sequencing data, taxonomic assignment was performed using MetaPhlAn (v1.7.7) (23) with default parameters.

### Gene Catalog Construction and Functional Annotations

The assembled genes of each sample were predicted using Prodigal (v3.02) (24). CD-HIT was used to cluster genes derived from all samples with a default identity of 0.95 and coverage of 0.9 (25). In order to analyze the relative abundance of unigenes in each sample, paired-end clean reads were mapped to unigenes using SOAPAligner (version 2.2.1) (26). Gene abundance was calculated based on the number of aligned reads and normalized to gene length, combined with grouping information. Venn diagrams were drawn based on the number of unique genes shared by two groups.

Diamond (version v0.8.15.77) (27) was used to search the protein sequences of the unigenes with the NR database, CAZy database, eggNOG database, and Kyoto Encyclopedia of Genes and Genomes (KEGG) database. The statistical significance threshold of the sequence alignment was set to 1e-5, and the sequence alignment length was set as no less than 60% of the reference gene protein length. Finally, the matched result with best hit scores was selected for annotation.

### Statistical Analysis of 16S rRNA Gene Sequencing and Metagenomic Sequencing Data

The Pearson coefficient was calculated by R stats package corr function based on the genus level abundance from 16S rRNA gene sequencing and metagenomic sequencing in the same sample. PCoA and NMDS analysis were performed using APE package in R, based on the Brary–Curtis matrix of either taxonomic abundance or selected unigene abundance. Additionally, Vegan package in R was used for ANOSIM analysis to analyze the differences between and within groups. Lefse analysis (http://huttenhower.sph.harvard.edu/galaxy) was used to characterize the differentially enriched microbes (linear discriminant analysis, LDA >2; *P*-value <0.05) in each group.

The differentially significantly enriched or depleted genes in each group were identified with the following criteria: (1) at least twofold changes between the Ob&H and Ob&T2D groups and (2) false discovery rate should be less than 0.1. The identified enriched or depleted genes in the Ob&T2D group were submitted to KEGG enrichment analysis, and the significantly enriched KEGG Ontology (KO) (*q*-value <0.05) was summarized in **Supplementary Table S3**.

### Random Forest Model Prediction

Random forest (28) analysis provided in the R package random forest was used to build the prediction model to identify the potential diagnostic biomarker genes between the Ob&H and Ob&T2D groups. The gene abundance profiles were calculated from metagenomic sequencing results. The top 300 depleted genes in the Ob&T2D group were filtered as prediction input variables. The important genes that contributed to prediction were identified *via* a 10-fold cross-validation procedure. The area under curve (AUC) index and receiver operating characteristic (ROC) analysis were used to predict the efficiency of the possible cutoff values of the tests.

## Results

### Overview of the Characteristics of the Cohort

**Figure 1:**
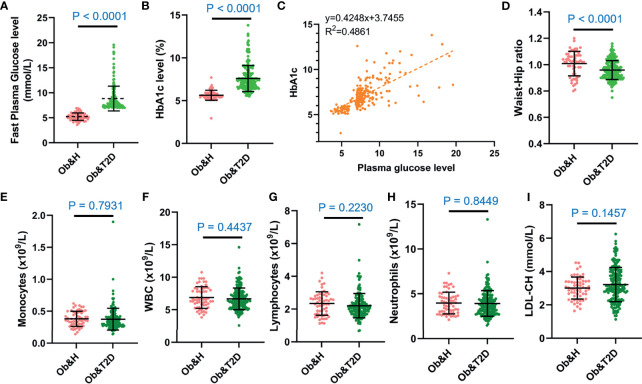
The overall characteristics of the study participants. **(A, B)** Dot plots display the fasting plasma glucose **(A)** and HbA1c levels **(B)** of the study participants. **(C)** Correlation between capillary plasma glucose level and HbA1c level. **(D)** Waist-to-hip ratio of the study participants. **(E–I)** Inflammation-related indices of the study participants: monocytes **(E)**, white blood cell **(F)**, lymphocytes **(G)**, neutrophils **(H)**, and low-density lipoprotein **(I)**. Ob&H, obese and healthy; Ob&T2D, obese and type 2 diabetes. Data are shown as means ± SD. All *P*-values were from Wilcoxon rank-sum tests.

We recruited 60 individuals with central obesity (male/female, 27:33) with normal fasting glucose level (“central obese healthy,” Ob&H, average fasting blood glucose level: 5.2 ± 0.7 mmol/L), 183 individuals with central obesity (male/female, 72:111) who were newly diagnosed as T2D and thus diabetes-treatment-naïve (“central obese T2D,” Ob&T2D, average fasting blood glucose level: 8.8 ± 2.5 mmol/L) (**Table 1**; **Figure 1A**). Similarly, the hemoglobin A1c (HbA1c) level in Ob&T2D (average: 7.6 ± 1.5%) is also significantly higher than in Ob&H individuals (average: 5.6 ± 0.6%) (**Table 1**; **Figure 1B**), in line with the positive correlation between HbA1c level with fasting blood glucose level (*R* ^2^ = 0.46) (**Figure 1C**). In addition to the expected alterations in blood glucose status, lower weight, height, body mass index, and waist circumference were observed in Ob&T2D, compared with the Ob&H group (**Table 1**; **Figure 1D**).

**Table 1:** The overall characteristics of the participants in this study.

Characteristics	Ob&H (*n* = 60)	Ob&T2D (*n* = 183)	*P*-value (Wilcoxon rank-sum test)
Sex (M/F)	27/33	72/111	
Fast plasma glucose (mmol/L)	5.2 ± 0.7	8.8 ± 2.5****	<0.0001
HbA1c (%)	5.6 ± 0.6	7.6 ± 1.5****	<0.0001
Weight (kg)	81.1 ± 17.1	73.4 ± 12.7***	0.0003
Height (cm)	164.0 ± 7.9	160.8 ± 7.8**	0.0066
BMI	30 ± 5	28 ± 4**	0.0092
Waist circumference (cm)	102.9 ± 10.1	97.1 ± 7.7****	<0.0001
Hip circumference (cm)	102.6 ± 10.0	101.2 ± 7.8^n.s.^	0.3413
Waist–hip ratio	1.01± 0.09	0.96 ± 0.07****	<0.0001

Plus–minus values are means ± SD. P-values were counted from Wilcoxon rank-sum tests.

M, male; F, female; HbA1c, hemoglobin A1c; BMI, body mass index; n.s., non-significant.

****P < 0.0001, ***P < 0.001, **P < 0.01.

The other clinical characteristics and metabolic biomarkers in the two groups are summarized in **Table 1** and **Supplementary Table S1**. No significant differences in inflammation-related markers, including monocytes, white blood cells, lymphocytes, neutrophils, or low-density lipoprotein, were observed between the Ob&H and Ob&T2D groups (**Figures 1E–I**; **Table 1**).

### Alterations in Gut Microbiota Composition Profiles in Ob&H and Ob&T2D Based on the 16S rRNA Gene Sequencing Data

Bacterial genomic DNA was isolated from the 243 stool samples for 16S rRNA (V3 and V4 regions) gene and metagenomic sequencing. The average number of raw paired reads per sample was 61,268 ± 16,703 reads for the 16S library of intestinal flora and 19,890,123 ± 2,854,537 reads from the metagenomic sequencing intestinal flora (**Supplementary Table S2**). Additionally, the rarefaction curves generated from the OTUs showed sufficient sequencing depth (**Supplementary Figure S1A**).

**Figure 2:**
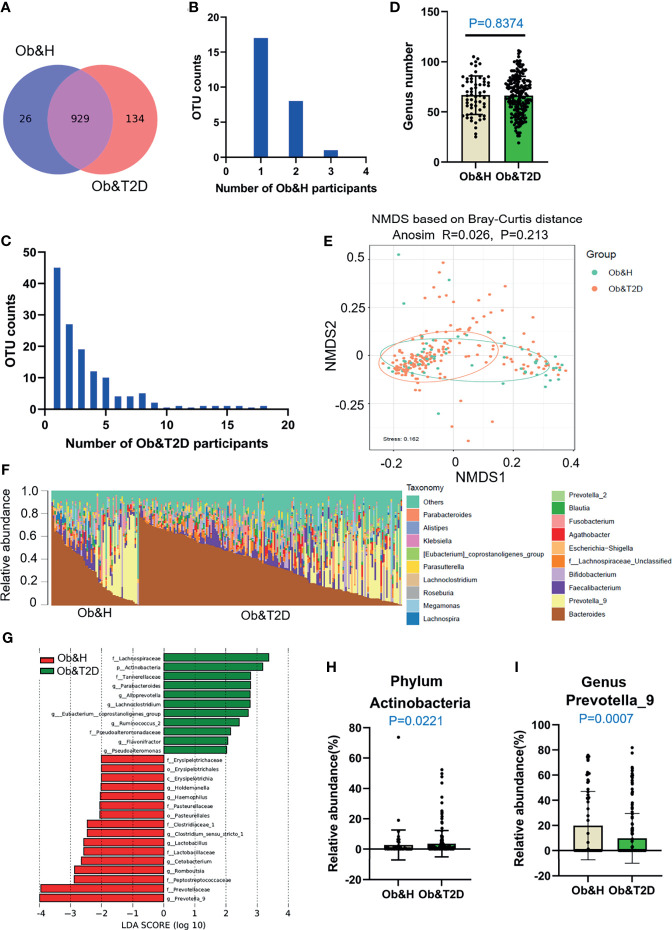
Differential gut microbiota analysis of obese and healthy (Ob&H) and obese and type 2 diabetes (Ob&T2D) patients. **(A)** Venn diagram of the observed operational taxonomic units (OTUs) in Ob&H and Ob&T2D. **(B, C)** Frequency distributions of the 26 Ob&H- **(B)** and 134 Ob&T2D-specific OTUs **(C)** in the study participants. **(D)** Estimate of richness analysis between the two groups at the genus level. Data are shown as means ± SD. **(E)** Nonmetric multidimensional scaling analysis of the gut microbiota based on the Bray–Curtis distance for Ob&H and Ob&T2D, ANOSIM, *R* = 0.026, *P* = 0.213. **(F)** Genus-level taxonomic abundances for gut 16S rRNA gene sequencing data. The top 20 abundant genera are annotated in the panel legend. Samples are ordered according to decreasing relative abundance of Bacteroides in the Ob&H and Ob&T2D groups. **(G)** Linear discriminant analysis (LDA) scores for the gut bacterial taxa differentially abundant between Ob&H and Ob&T2D. Red bars with LDA score greater than 2 indicate that the taxa were enriched in Ob&H, and green bars with LDA score greater than 2 indicate that the taxa were enriched in Ob&T2D. **(H, I)** Relative abundance of phylum *Actinobacteria* **(H)** and genus *Prevotella_9* **(I)** in Ob&H and Ob&T2D. Data are shown as means ± SD. All *P-*values are from Wilcoxon rank-sum tests.

As shown in **Figure 2A**, 929 OTUs (87% of OTUs in Ob&T2D and 97% of the OTUs in Ob&H) were shared by both groups. Furthermore, 26 and 134 specific OTUs were observed in the Ob&H and Ob&T2D groups, respectively (**Figure 2A**). The 26 Ob&H group-specific OTUs were randomly distributed in the Ob&H participants, most of which were found only in one or two individuals. However, 31 of 134 (23%) Ob&T2D OTUs existed at least in five individuals, and eight (6%) of 134 were shared by at least 10 individuals (**Figures 2B, C** **)**. Further taxonomy annotations of those eight OTUs revealed that they were from the genera *Ruminiclostridium*, *Pseudoalteromonas*, *Alloprevotella*, and *Bacteroides*, suggesting the preferential colonization of these kinds of gut microbiota in the Ob&T2D individuals. Nevertheless, species richness at the genus level was comparable between the Ob&H and Ob&T2D groups (*P* = 0.8374) (**Figure 2D**). Furthermore, the NMDS analysis (**Figure 2E**) and the PCoA (**Supplementary Figure S1B**) of the microbial communities based on the Bray–Curtis distances indicated similar gut microbiota communities from these two cohorts (ANOSIM, R = 0.026, *P* = 0.213). The Shannon index (**Supplementary Figure S2A**) and Chao1 (**Supplementary Figure S2B**), which account for the evenness and abundance of species, of the Ob&H group showed no significant changes compared with the Ob&T2D group. Furthermore, the phylogenetic diversity (PD) index, which measures the degree of evolutionary divergence, was also comparable between the two groups (**Supplementary Figure S2C**). This agrees with the previous finding that the alpha diversity was not significantly reduced between obese individuals with or without T2D (29). Since obesity is a strong risk factor for developing T2D (3) and the participants were newly diagnosed as T2D in our Ob&T2D cohort, they are expected to share similar microbiome features with regards to alpha diversity.

Although there is no significant difference in alpha diversity between the two groups, the bacterial community profiles at the genus level showed different patterns among individuals (**Figure 2F**). The LDA distribution diagram analysis (LDA score >2) showed a clear alteration in the microbiota characterized by higher *Actinobacteria* levels in Ob&T2D individuals (from 2.73 ± 9.88% in Ob&H to 3.58 ± 8.69% in Ob&T2D, Wilcoxon rank-sum test *p*-value = 0.0221) (**Figures 2G, H** **)**. The genera *Parabacteroides*, *Alloprevotella*, *Lachnoclostridium*, *Eubacterium_coprostanoligenes*, *Ruminococcus 2*, *Flavonifractor*, and *Pseudoalteromonas* were markedly enriched (LDA score >2) in the Ob&T2D group, while the genera *Prevotella 9*, *Romboutsia*, *Cetobacterium*, *Lactobacillus*, *Clostrium_sensu_stricto*, *Haemophilus*, and *Holdemanella* were more abundant (LDA score >2) in the Ob&H group (**Figures 2G, I** **)**, indicating that these kinds of differential genera might be involved in the gastrointestinal status transition from central obesity to central obese diabetes. The other abundant phyla, such as *Bacteroidetes*, *Firmicutes*, and *Proteobacteria* (**Supplementary Figure S3**), and genera, such as *Bacteroides*, *Faecalibacterium*, and *Bifidobacterium* (**Supplementary Figure S4**), were comparable between the groups. The abovementioned analyses altogether suggested that the dysbiosis of gut microecology may contribute to the development of T2D from obesity.

### Metagenomic Sequencing Revealed an Accumulation of Opportunistic Pathogens in Ob&T2D Patients

**Figure 3:**
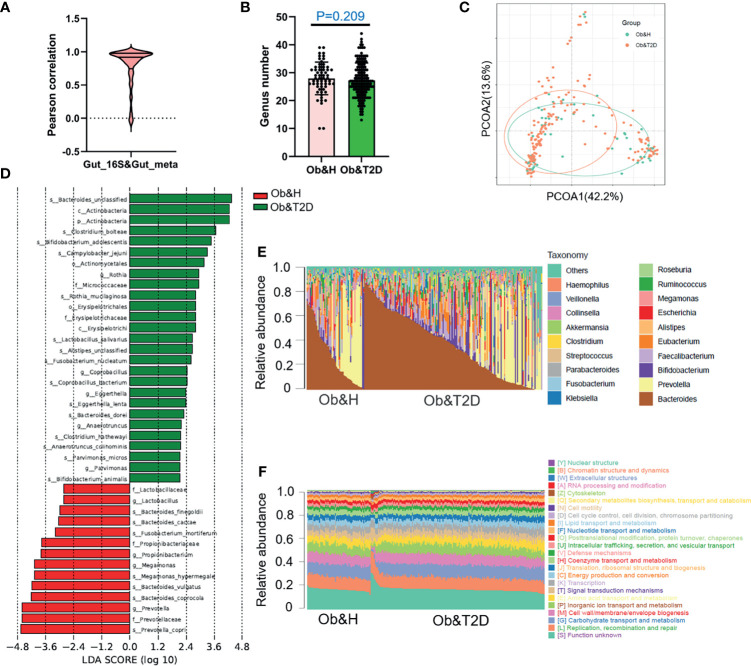
Gut microbiota divergence analysis in obese and healthy (Ob&H) and obese and type 2 diabetes (Ob&T2D) based on metagenomic sequencing data. **(A)** Violin plot displaying Pearson correlation between gut 16S rRNA gene sequencing and metagenomic sequencing data. **(B)** Estimate of richness analysis between the two groups at the genus level. Data are shown as means ± SD. **(C)** Principal coordinate analysis (PCoA) of the microbiota based on the Bray–Curtis distance metrics for Ob&H and Ob&T2D. ANOSIM, *R* = 0.107, *P* = 0.001. **(D)** Genus-level taxonomic abundances for gut metagenomic sequencing data. The top 20 abundant genera are annotated in the panel legend. The samples are ordered according to decreasing relative abundance of *Bacteroides* in the Ob&H and Ob&T2D groups. **(E)** Predicted eggNOG orthologous functional annotations of gut microbial unique genes from metagenomic data. **(F)** The linear discriminant analysis (LDA) scores for the gut bacterial taxa are differentially abundant between Ob&H and Ob&T2D. Red bars with LDA score greater than 2 indicate that the taxa were enriched in Ob&H, and green bars with LDA score greater than 2 indicate that the taxa were enriched in Ob&T2D.

To investigate the potential effect of the gut microbiome on gastrointestinal symptoms in Ob&T2D patients and to address whether gut microbial changes at the genera level, species level, or both are associated with progressing from central obesity to T2D, metagenomic sequencing was applied to these fecal samples. As expected, the relative genus abundances from metagenomic sequencing were highly correlated with those from 16S rRNA gene sequencing (**Figure 3A**). The genus numbers were comparable between the two groups, consistent with the 16S rRNA gene sequencing data (**Figure 2B** and **Figure 3B**). The PCoA analysis based on the Bray–Curtis distance matrix revealed small differences in microbial composition between the Ob&H and Ob&T2D groups at the genus level (ANOSIM, *R* = 0.107, *P* = 0.001) (**Figure 3C**). Next, we compared the bacterial profile differences between the Ob&H and Ob&T2D groups. Consistent with 16S rRNA gene sequencing data at the phylum and genus levels, *Actinobacteria* was markedly enriched in the Ob&T2D group, and the genus *Prevotella* was more abundant in the Ob&H group (**Figure 3D**). A total of 22 species from various genera showed differential enrichment between the two groups. Seven species (for example, *Prevotella_copri*, *Fusobacterium_mortiferum*, and *Bacteroides_coprocola*) from five genera were enriched in the Ob&H group, and 15 species from 12 genera were enriched in the Ob&T2D group (**Table 2**). In contrast to the Ob&H group, many of the Ob&T2D-enriched species were opportunistic pathogens, such as *Eggerthella lenta*, *Clostridium hathewayi*, *Clostridium bolteae*, and *Parvimonas micros* (**Figure 3D**; **Table 2**), which have previously been reported to cause or be correlated with human abdominal infectious diseases (30, 31). Although the decreased abundance of butyrate producer microbiota has been reported to be associated with the development of T2D (9, 10), we did not find any significant difference in several representative butyrate producer microbiota (*Eubacterium rectale*, *Roseburia intestinalis*, *Roseburia inulinivorans*, *Eubacterium hallii*, and *Faecalibacterium prausnitzii*) abundance between the Ob&H and Ob&T2D groups (**Supplementary Figure S5**). This suggested that those butyrate producers may have already been depleted in centrally obese patients.

**Table 2:** List of Ob&T2D- and Ob&H-enriched species from metagenomic sequencing data.

Species	Enriched group	LDA	*P*-value
**Ob&T2D-enriched**			
*s:Bacteroides_dorei*	Ob&T2D	2.29	0.04
*s:Clostridium_hathewayi*	Ob&T2D	2.17	0.04
*s:Rothia_mucilaginosa*	Ob&T2D	2.78	0.02
*s:Parvimonas_micros*	Ob&T2D	2.13	0.03
*s:Campylobacter_jejuni*	Ob&T2D	3.31	0.04
*s:Clostridium_bolteae*	Ob&T2D	3.65	0.01
*s:Coprobacillus_bacterium*	Ob&T2D	2.42	0.01
*s:Fusobacterium_nucleatum*	Ob&T2D	2.61	0.03
*s:Bifidobacterium_animalis*	Ob&T2D	2.12	0.01
*s:Bifidobacterium_adolescentis*	Ob&T2D	3.46	0.01
*s:Anaerotruncus_colihominis*	Ob&T2D	2.15	0.01
*s:Eggerthella_lenta*	Ob&T2D	2.38	0.01
*s:Lactobacillus_salivarius*	Ob&T2D	2.66	0.02
*s:Alistipes_unclassified*	Ob&T2D	2.66	0.04
*s:Bacteroides_unclassified*	Ob&T2D	4.36	0.00
**Ob&H-enriched**			
*s:Prevotella_copri*	Ob&H	4.66	0.00
*s:Bacteroides_finegoldii*	Ob&H	2.98	0.04
*s:Megamonas_hypermegale*	Ob&H	4.08	0.01
*s:Bacteroides_coprocola*	Ob&H	4.20	0.00
*s:Bacteroides_vulgatus*	Ob&H	4.18	0.02
*s:Fusobacterium_mortiferum*	Ob&H	3.17	0.02
*s:Bacteroides_caccae*	Ob&H	3.03	0.04

Interestingly, in our study, although the relative abundance of each microbiota varies greatly among individuals (**Figure 3E**), their functional compositions were consistent with each other, regardless of the Ob&H or Ob&T2D groups (**Figure 3F**), which was also observed in other study populations (29). The genes involved in replication, recombination and repair, carbohydrate transport and metabolism, cell wall/membrane/envelope biogenesis, inorganic ion transport and metabolism, and amino acid transport and metabolism represented the most abundant five eggNOG-annotated categories in the two groups (**Figure 3F**). This result implied that the genic pathway composition is shaped by gut microbiota communities rather than the microbiota population itself, which matters in the gastrointestinal ecosystem.

### The Depletion of Gut Microbial Carbohydrate Metabolism Is Associated With the Development of T2D From Central Obesity

**Figure 4:**
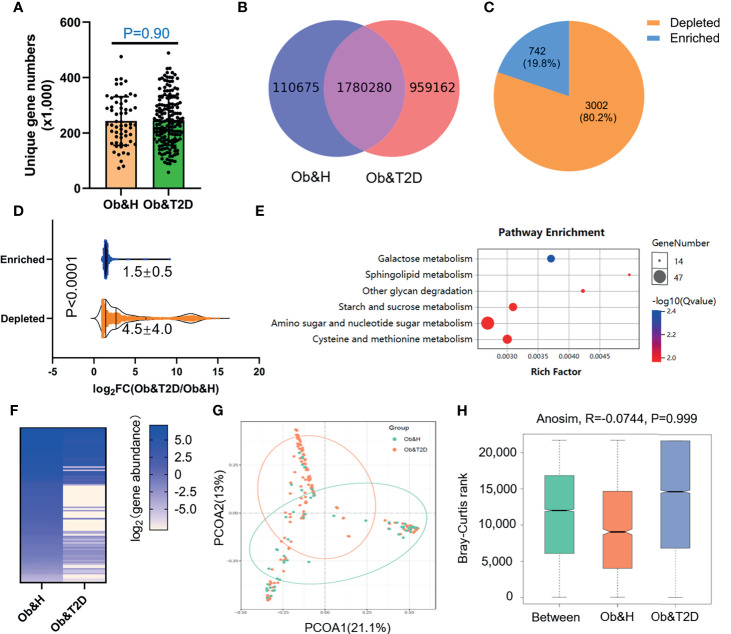
Differential gut microbial gene functions annotation on Kyoto Encyclopedia of Genes and Genomes (KEGG) in obese and healthy (Ob&H) and obese and type 2 diabetes (Ob&T2D). **(A)** Box plots show the number of observed gut microbial unique genes in Ob&H and Ob&T2D. Data are shown as means ± SD. The *P*-value is from Wilcoxon rank-sum tests. **(B)** Venn diagrams demonstrate the number of unique genes shared between Ob&H and Ob&T2D. **(C)** The pie chart displays the number of enriched or depleted genes (filtering criteria: *q*-value <0.1, at least twofold change) in Ob&T2D compared with Ob&H. **(D)** Violin plots display the changing fold of enriched or depleted genes. Data are shown as means ± SD. The *P*-value is from Wilcoxon rank-sum tests. **(E)** Representative gene ontology enrichment (*q*-value <0.05) analysis of 3,002 depleted genes in Ob&T2D. **(F)** Heat map depicting the log_2_ (gene abundance) of 96 representative genes involved in the enriched KEGG pathways in **(E)**. **(G)** Principal coordinate analysis based on the Bray–Curtis distance of 96 depleted gene abundance between Ob&H and Ob&T2D. **(H)** Comparison of the enriched KEGG orthologous between Ob&H and Ob&T2D. ANOSIM, *R* = −0.0744, *P* = 0.999.

To further compare the functional bacteria gene alteration during the development of Ob&T2D from obesity, we analyzed gut microbial functions at the gene level across the two groups in our study cohort. A total of 2,850,117 genes were predicted in this study, with an average of 243,400 and 245,900 genes predicted in the Ob&H and Ob&T2D groups, respectively (**Figures 4A, B** **)**. Notably, nearly one million unique genes were specifically detected in the Ob&T2D group, in line with previous findings that 134 OTUs were specifically detected in the Ob&T2D group (**Figure 2A**). Furthermore, we identified 3,744 differentially enriched (fold change ≥2, *q*-value <0.1) unique genes between two groups, the vast majority [3,002 (80.2%) of the total of 3,744 genes] of which were evidently depleted in Ob&T2D, and only a small subset [742 (19.8%) of the total of 3,744 genes] of the genes was significantly enriched in Ob&T2D (**Figure 4C** and **Supplementary Table S3**). Meanwhile, the depletion fold (average fold change = 22.6) was significantly higher than that of enrichment (average fold change = 2.8) (**Figure 4D**), resulting in an overall depletion of the gut micro-ecologic gene abundances during the development of T2D from obesity. Next, the KO enrichment analysis of 3,002 depleted and 742 enriched genes in Ob&T2D revealed that several sugar and amino acid metabolic pathways were significantly enriched (*q*-value <0.05) in the 3,002 depleted genes in the Ob&T2D subgroup (**Figure 4E** and **Supplementary Table S4**). Those depleted pathways included, for example, the metabolism of galactose, sphingolipid, glycan, starch and sucrose, amino sugar and nucleotide sugar, and cysteine and methionine (**Figure 4E** and **Supplementary Table S4**). However, no significantly enriched KO was identified in the 742 enriched genes in the Ob&T2D cohort (**Supplementary Table S4**). Our study together revealed a profound genic composition alteration during the transition from central obesity to T2D. Our results also suggested that the dysfunction in several sugar- and amino acid-related metabolism pathways (especially those of galactose, glycan, starch, and sucrose metabolism) in the gut microbial communities may be highly associated with this transition process.

### Gut Microbial Gene Profiles-Based Screening of Central Obese Individuals With a High Risk of Developing T2D

**Figure 5:**
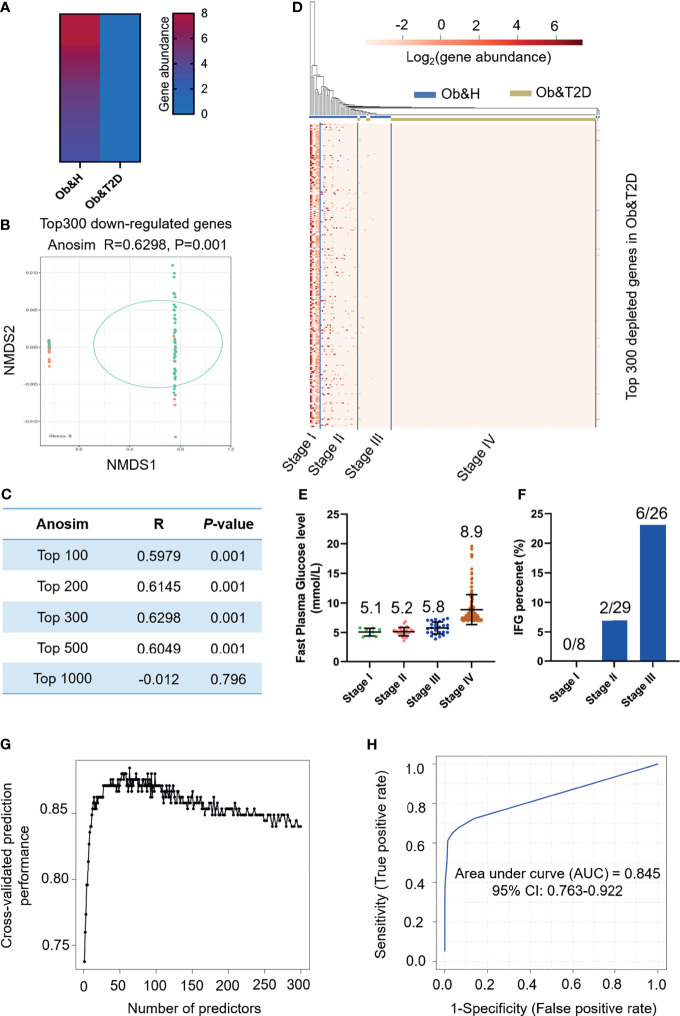
Distinguishing obese and type 2 diabetes (Ob&T2D) from obese and healthy (Ob&H) based on differentially enriched gut microbial genes. **(A)** Heat map depicting the gene abundance of the top 300 [rank by log_2_ (fold change)] Ob&T2D-depleted genes in both groups. **(B)** Nonmetric multidimensional scaling based on the Bray–Curtis distance of the top 300 depleted gene abundance in Ob&T2D. ANOSIM, *R* = 0.6298, *P* = 0.001. **(C)** Listing of ANOSIM analysis R, *P*, and stress values based on the abundance of top *N* (*N* = 100, 200, 300, 500, and 1,000) depleted genes in Ob&T2D. **(D)** Heat map displaying the gene abundance of the top 300 [rank by log_2_ (fold change)] Ob&T2D-depleted genes in each participant. The samples were grouped into four stages (stages I–IV) according to their gene abundance. **(E)** Dot plots presenting the fasting plasma glucose levels in the individuals of each stage. The number of each stage is as follows: stage I (*n* = 8), stage II (*n* = 29), stage III (*n* = 26), and stage IV (*n* = 159). **(F)** Percentage of people with impaired fasting plasma glucose levels (6.1 mmol/L<fasting plasma glucose level<7 mmol/L). **(G)** Classification performance of a random forest model using the abundance of the top 300 depleted genes as assessed by R random forest package. **(H)** Receiver operating characteristic curve displaying the classification for Ob&H and Ob&T2D using gene abundance profiles. AUC, area under curve.

Next, we employed the gut microbiota gene profile-based method to identify the potential diagnostic biomarkers for T2D status in obese populations. Firstly, we performed PCoA analysis based on the abundances of the 96 depleted genes involved in significantly enriched KEGG pathways (galactose metabolism, sphingolipid metabolism, glycan degradation, starch and sucrose metabolism, amino sugar and nucleotide sugar metabolism, and cysteine and methionine metabolism) (**Figures 4E, F**; **Supplementary Table S5**). The results showed that the two groups were indistinguishable based on the 96 depleted gene abundances as mentioned above (**Figure 4G**), and the differences within groups were larger than that of between groups (ANOSIM, *R* = −0.0744, *P* = 0.999) (**Figure 4H**). This may be explained because the abundance differences of these 96 depleted genes between Ob&H and Ob&T2D were not large enough to set them apart. To further test this hypothesis, we conducted a series of PCoA, NMDS, and ANOSIM analyses based on the abundance of the top *N*-ranked (*N* = 100, 200, 300, 400, 500, and 742 for enriched genes and *N* = 100, 200, 300, 400, 500, and 1,000 for depleted genes, ranked by the changing folds of gene abundance between the Ob&T2D and Ob&H groups) enriched or depleted genes in the Ob&T2D subgroup. As expected, the PCoA results based on the abundance of the top *N*-ranked enriched genes in Ob&T2D, whose changing folds are much smaller than those of depleted genes (**Figure 4D**), showed no distinguishing effects between the two groups (**Supplementary Figure S6**). Interestingly, Ob&T2D and Ob&H can be obviously separated into two different clusters based on the abundance of the top 300 depleted genes in the Ob&T2D subgroup (ANOSIM *R* = 0.6298, *P* = 0.001) (**Figures 5A, B** **)**. A similar separation was achieved when we performed the same analyses based on the abundance of the top 100, 200, 400, and 500 genes, but not the top 1,000 depleted genes, and the maximum differences between groups were achieved when using the top 300 depleted genes for ANOSIM analysis (**Figure 5C**; **Supplementary Figure S7**), supporting the hypothesis that the top depleted gene sets are better biomarkers than the enriched ones in distinguishing Ob&H and Ob&T2D. We then divided all the samples into four stages, stage I to stage IV, with decreasing abundances of these 300 depleted genes (**Figure 5D**). Interestingly, we found that their fasting plasma glucose level negatively correlated, and the percentage of people with impaired fasting glucose (between 6.1 and 7.0 mmol/L) was positively correlated with the abundance of those 300 depleted genes (**Figures 5E, F** **)**. These results supported that the gut microbiota gene profiles can be used as potential diagnostic biomarkers to predict the central obese individuals with high risks of developing T2D.

We then performed KEGG enrichment analysis by using these top 300 depleted genes in the Ob&T2D group. Similarly, genes that were involved in glycan degradation, sphingolipid metabolism, and galactose metabolism were also enriched as observed in the 3,002 depleted genes (**Supplementary Table S6**). In addition, numerous other pathways, including nucleotide excision repair and glycosaminoglycan degradation, were also enriched (*q*-value <0.05) (**Supplementary Table S6**). A further unique gene annotation revealed that 60% (180 of 300) of the most significantly depleted genes are from genus *Prevotella*, in line with the depletion of genus *Prevotella* in the Ob&T2D group (**Figures 2G**, **3D**). Several species, such as *Prevotella* sp. *CAG:520* (42 of 300), *Prevotella stercorea* (37 of 300), and *Prevotella pectinovora* (nine of 300), were also enriched (**Supplementary Table S3**). Therefore, the disturbance of those pathways in the representative species may collectively play a role in the progression from central obesity to T2D.

To further narrow the potential diagnostic biomarkers to predict Ob&T2D status, we developed a random forest classifier model based on the abundance profiles of the top 300 depleted genes. The optimal model utilized 63 marker genes which provided the best discriminatory power (**Figure 5G**). These genes in the optimal model were mostly from genus *Bacteroides* (15 of 63) and *Prevotella* (13 of 63) (**Supplementary Table S7**). The ROC curve was obtained based on the 63 optimal markers, achieving an AUC value of 0.845 with 95% CI of 0.763–0.922 (**Figure 5H**). Therefore, it is concluded that the prediction model showed a high discriminatory power to predict Ob&T2D status.

## Discussions

This study examined the gut microbial profiles of central obese individuals with or without T2D to dissect the potential gut microbial features associated with progressing from central obesity to T2D. Here we have found an alteration in the gut microbiota, especially at the gene functional level, in individuals with diabetes-treatment-naïve obese T2D compared with obese individuals. Furthermore, we identified a group of genes from the gut microbiota community involved in carbohydrate-related metabolism that can be used as potential diagnostic biomarkers to predict T2D status among the obese population.

As expected, our results showed that the microbial profiles were quite similar between the central obese individuals with or without T2D in alpha (Shannon index and Chao1) and beta diversity indices (PD), which is in line with the previous finding (29). Our further observation on the colonization of opportunistic pathogens, such as *Eggerthilla lenta*, *Clostridium hathewayi*, and *Clostridium bolteae*, in the gut of T2D is also in good agreement with previous studies (12). We extended those previous findings by showing that it is independent of central obesity or medical treatment, two major confounding factors affecting the gut microbiota (11, 16). Although several other kinds of opportunistic pathogens, such as *Escherichia coli*, were also reported to accumulate in the gut, inducing obesity and insulin resistance (12), our analyses revealed that the opportunistic pathogens colonized in the gut of Ob&T2D vary in each participant, and the types of colonized species are also different from previously reported species (10, 12, 32, 33). This may be due to the particular types of pathogens that our volunteers encountered. However, it is conceivable that the dysbiotic gut microbiome may causatively contribute to T2D development. The mechanism underlying the opportunistic pathogen colonization in the gut environment upon onset of T2D from central obesity remains unclear. Thus, further studies are needed to dissect its pathophysiology.

Our results also revealed profound genic alterations between the two groups. Thousands of genes involved in the metabolism of galactose, sphingolipid, glycan, starch, and sucrose were depleted in the central obese individuals with T2D. This finding suggests that the capabilities of sugar metabolism in gut microbiota are greatly impaired in central obese T2D individuals. It has been widely reported that short-chain fatty acids (SCFAs), which are the primary metabolites produced by gut bacterial fermentation of dietary fiber in the gastrointestinal tract, play an essential role in regulating intestinal homeostasis, and their anti-inflammatory activities have also been well documented (34, 35). Different kinds of carbohydrates are sources for SCFA biogenesis (36); therefore, we proposed that gut microbial sugar metabolism dysfunction may lead to a shortage of SCFA production, resulting in low-grade inflammation in the gut microenvironment. Low sugar usage efficiency by gut bacteria, in turn, also strengthens the burden of the human host to absorb and digest the remaining sugar, and accumulating evidence has demonstrated that high-sugar diets promote the development of T2D by inducing weight gain and insulin resistance (37, 38). In this scenario, our study provided a potential prebiotics development or transplantation strategy to prevent and/or delay T2D development from central obesity.

Furthermore, we identified hundreds of genes whose abundance is correlated with glycemic level (**Figure 5** and **Supplementary Figure S8**). Thus, these can be used as potential gene markers to predict central obese individuals with a high risk of developing T2D. Several of these genes are enriched in the metabolism of different carbohydrates and nucleotide excision repair. Nevertheless, the biological functions and their potential roles in T2D development of other genes remain to be elucidated. These differentially enriched marker genes can also be the potential targets for probiotic bacterial engineering to prevent or manage T2D.

## Data Availability Statement

The data presented in the study are deposited in the China National GeneBank Sequence Archieve (CNSA)(https://db.cngb.org/cnsa/), accession number CNP0002048.

## Ethics Statement

The studies involving human participants were reviewed and approved by the Ethics Committee of Suzhou Municipal Hospital, Suzhou, China. The patients/participants provided their written informed consent to participate in this study.

## Author Contributions

HW, YH, RJ, MH, WC, and YJ conceived the project and designed the experiments. RJ, LQ, MH, XY, QL, and HY collected the experimental samples. RJ, MH, YH, and HW performed the 16S rRNA gene sequencing and metagenomic sequencing and bioinformatic data analysis with input from LY, YC, XW, WC, and YJ. RJ, MH, HW, and YH wrote the manuscript with input from all authors. All authors contributed to the article and approved the submitted version.

## Funding

This work was supported by the National Key Research and Development Program of China (grant 2016YFE0115900 to GENEWIZ), the Key Medical Disciplines in Jiangsu Province (grant no. ZDXKC2016007 to YH), the Science, Education for Health Foundation of Suzhou for Youth (grant no. KJXW2019033 to YH) and GENEWIZ Inc., Suzhou, China. The funders were not involved in the study design, collection, analysis, and interpretation of data, the writing of this article or the decision to submit it for publication.

## Conflict of Interest

RJ, LQ, HY, LY, XW, YC, YJ, and HW are fulltime employees of GENEWIZ Inc. WC was a fulltime employee of GENEWIZ Inc., Suzhou, China.

The remaining authors declare that the research was conducted in the absence of any commercial or financial relationships that could be construed as a potential conflict of interest.

## Publisher’s Note

All claims expressed in this article are solely those of the authors and do not necessarily represent those of their affiliated organizations, or those of the publisher, the editors and the reviewers. Any product that may be evaluated in this article, or claim that may be made by its manufacturer, is not guaranteed or endorsed by the publisher.

## Supplementary Material

Supplementary Figure 1 Gut microbiota analysis in Ob&H and Ob&T2D according to the 16S rRNA gene sequencing data. **(A)** Rarefaction curves of gut 16S rRNA gene sequencing reads. **(B)** Principal coordinate analysis (PCoA) of the gut microbiota based on the Bray-Curtis distance for Ob&H and Ob&T2D.

Supplementary Figure 2 Alpha and beta diversity indices of gut microbiota genus for Ob&H and Ob&T2D. Estimate of Shannon index **(A)**, Chao1 **(B)** and phylogenetic diversity index **(C)**. Data are shown as means ± SD. All P values are from Wilcoxon rank-sum tests. Ob&H: n = 60, Ob&T2D: n = 183.

Supplementary Figure 3 Relative abundance of indicated gut microbial phylum in Ob&H and Ob&T2D. **(A-C)**, Relative abundance of phylum Bacteroidetes **(A)**, Firmicutes **(B)** and Proteobacteria **(C)** in Ob&H and Ob&T2D gut microbiota. Data are shown as means ± SD. All P-values are from Wilcoxon rank-sum tests.

Supplementary Figure 4 Relative abundance of indicated gut microbial genera in Ob&H and Ob&T2D. **(A-C)**, Relative abundance of genus Bacteroides **(A)**, Faecalibacterium **(B)** and Bifidobacterium **(C)** in Ob&H and Ob&T2D gut microbiota. Data are shown as means ± SD. All P-values are from Wilcoxon rank-sum tests.

Supplementary Figure 5 Relative abundance of representative butyrate producer microbiota in Ob&H and Ob&T2D. **(A–E)** Relative abundance of specie Eubacterium rectale **(A)**, Eubacterium hallii **(B)**, Faecalibacterium prausnitzii **(C)**, Roseburia intestinalis **(D)** and Roseburia inulinivorans **(E)** in Ob&H and Ob&T2D. All P values are from Wilcoxon rank- sum tests.

Supplementary Figure 6 NMDS analysis based on the Bray-Curtis distance of top-ranked depleted gene abundance in Ob&T2D. **(A–D)**, NMDS analysis based on the Bray-Curtis distance of top N (N = 100, 200, 500 and 1000) depleted genes abundance in Ob&T2D. Anosim R and P values are shown as indicated.

Supplementary Figure 7 NMDS analysis based on the Bray-Curtis distance of top-ranked enriched gene abundance in Ob&T2D. **(A–D)**, NMDS analysis based on the Bray-Curtis distance of top N (N = 100, 200, 300 and 500) enriched genes abundance in Ob&T2D. Anosim R and P values are shown as indicated.

Supplementary Figure 8 Redundancy analysis(RDA) illustrating the relationship between clinal characteristics and microbial gene community. RDA analysis based on the abundance of top 300 depleted genes in Ob&T2D illustrating the relationship between clinical indices and microbial gene community. Glu: glucose level; HbA1c: Hemoglobin A1c; Mono: monocyte; ANC, neutrophils; WBC, white blood cell; LYMPH, ymphocyte.
