# Supplementary figures and images for: The Depletion of Carbohydrate Metabolic Genes in the Gut Microbiome Contributes to the Transition From Central Obesity to Type 2 Diabetes
Source: Front Endocrinol (Lausanne). 2021 Oct 22;12:747646. doi: 10.3389/fendo.2021.747646 (PMC8569854; doi:10.3389/fendo.2021.747646)

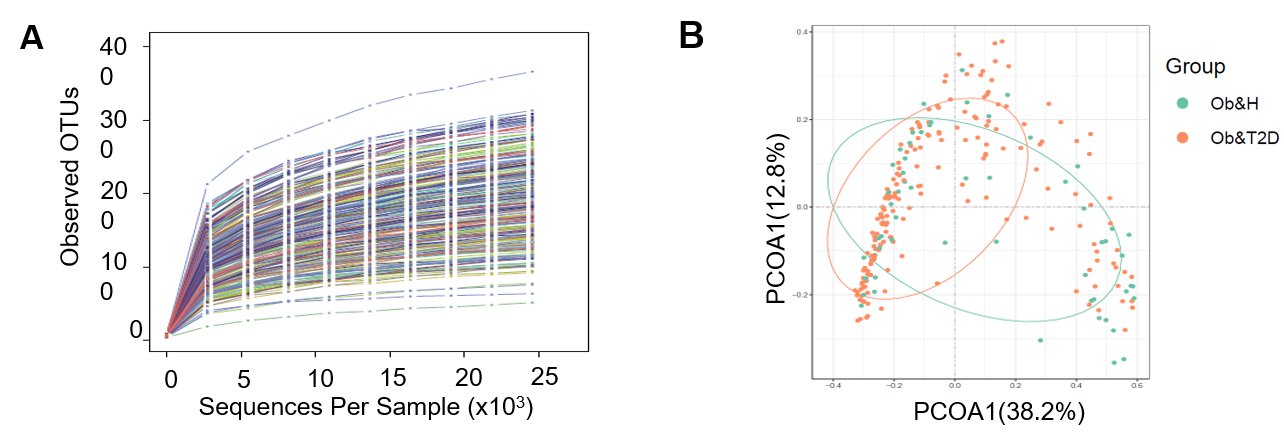

Supplement: Supplementary Figure 1 — Gut microbiota analysis in Ob&H and Ob&T2D according to the 16S rRNA gene sequencing data. (A) Rarefaction curves of gut 16S rRNA gene sequencing reads. (B) Principal coordinate analysis (PCoA) of the gut microbiota based on the Bray-Curtis distance for Ob&H and Ob&T2D. [file Image_1.tif]

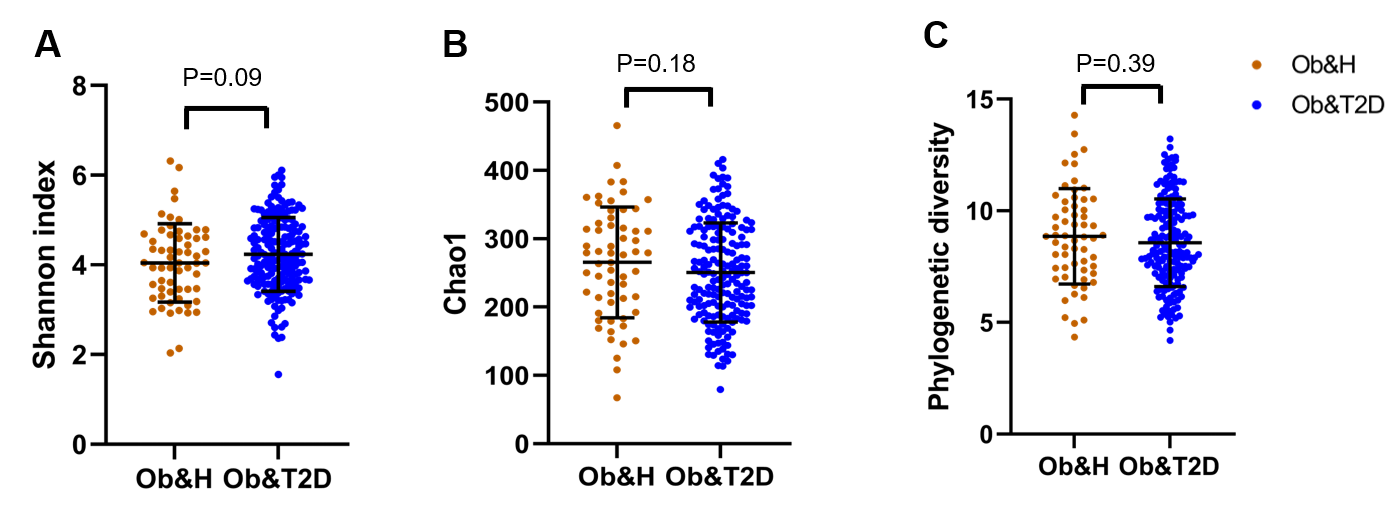

Supplement: Supplementary Figure 2 — Alpha and beta diversity indices of gut microbiota genus for Ob&H and Ob&T2D. Estimate of Shannon index (A), Chao1 (B) and phylogenetic diversity index (C). Data are shown as means ± SD. All P values are from Wilcoxon rank-sum tests. Ob&H: n = 60, Ob&T2D: n = 183. [file Image_2.tif]

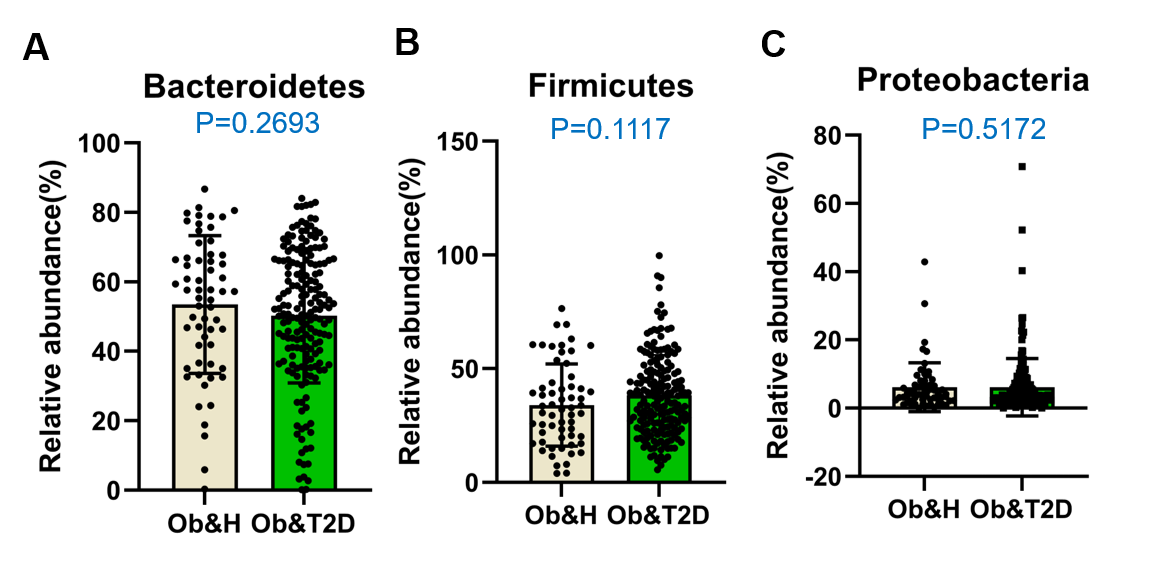

Supplement: Supplementary Figure 3 — Relative abundance of indicated gut microbial phylum in Ob&H and Ob&T2D. (A-C), Relative abundance of phylum Bacteroidetes (A), Firmicutes (B) and Proteobacteria (C) in Ob&H and Ob&T2D gut microbiota. Data are shown as means ± SD. All P-values are from Wilcoxon rank-sum tests. [file Image_3.tif]

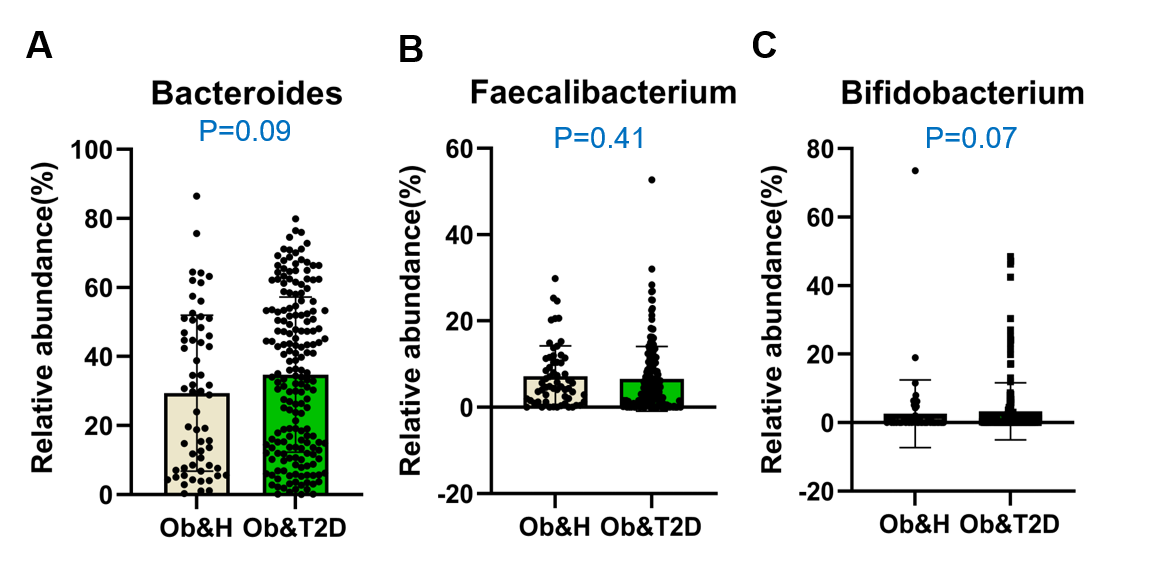

Supplement: Supplementary Figure 4 — Relative abundance of indicated gut microbial genera in Ob&H and Ob&T2D. (A-C), Relative abundance of genus Bacteroides (A), Faecalibacterium (B) and Bifidobacterium (C) in Ob&H and Ob&T2D gut microbiota. Data are shown as means ± SD. All P-values are from Wilcoxon rank-sum tests. [file Image_4.tif]

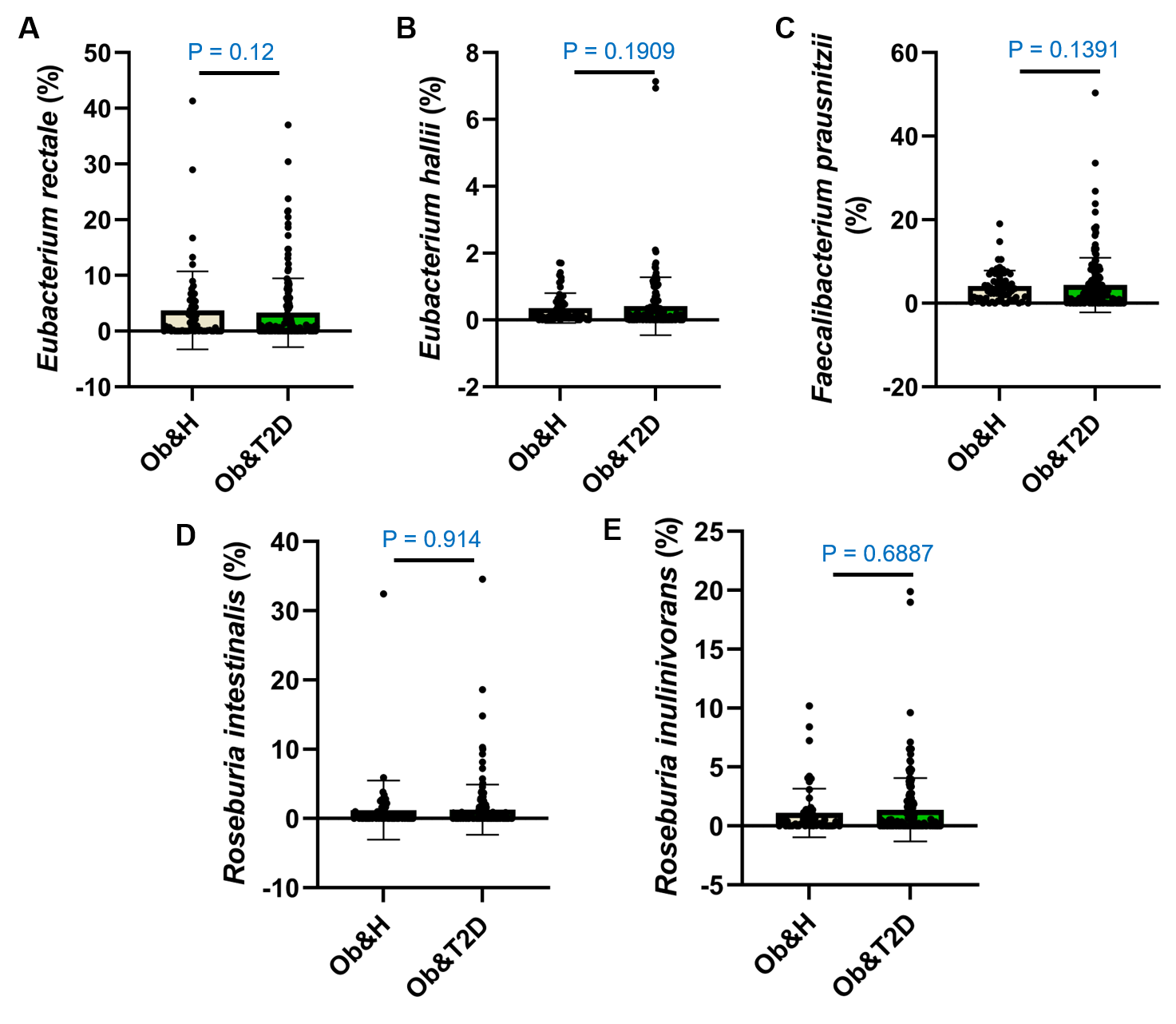

Supplement: Supplementary Figure 5 — Relative abundance of representative butyrate producer microbiota in Ob&H and Ob&T2D. (A–E) Relative abundance of specie Eubacterium rectale (A), Eubacterium hallii (B), Faecalibacterium prausnitzii (C), Roseburia intestinalis (D) and Roseburia inulinivorans (E) in Ob&H and Ob&T2D. All P values are from Wilcoxon rank- sum tests. [file Image_5.tif]

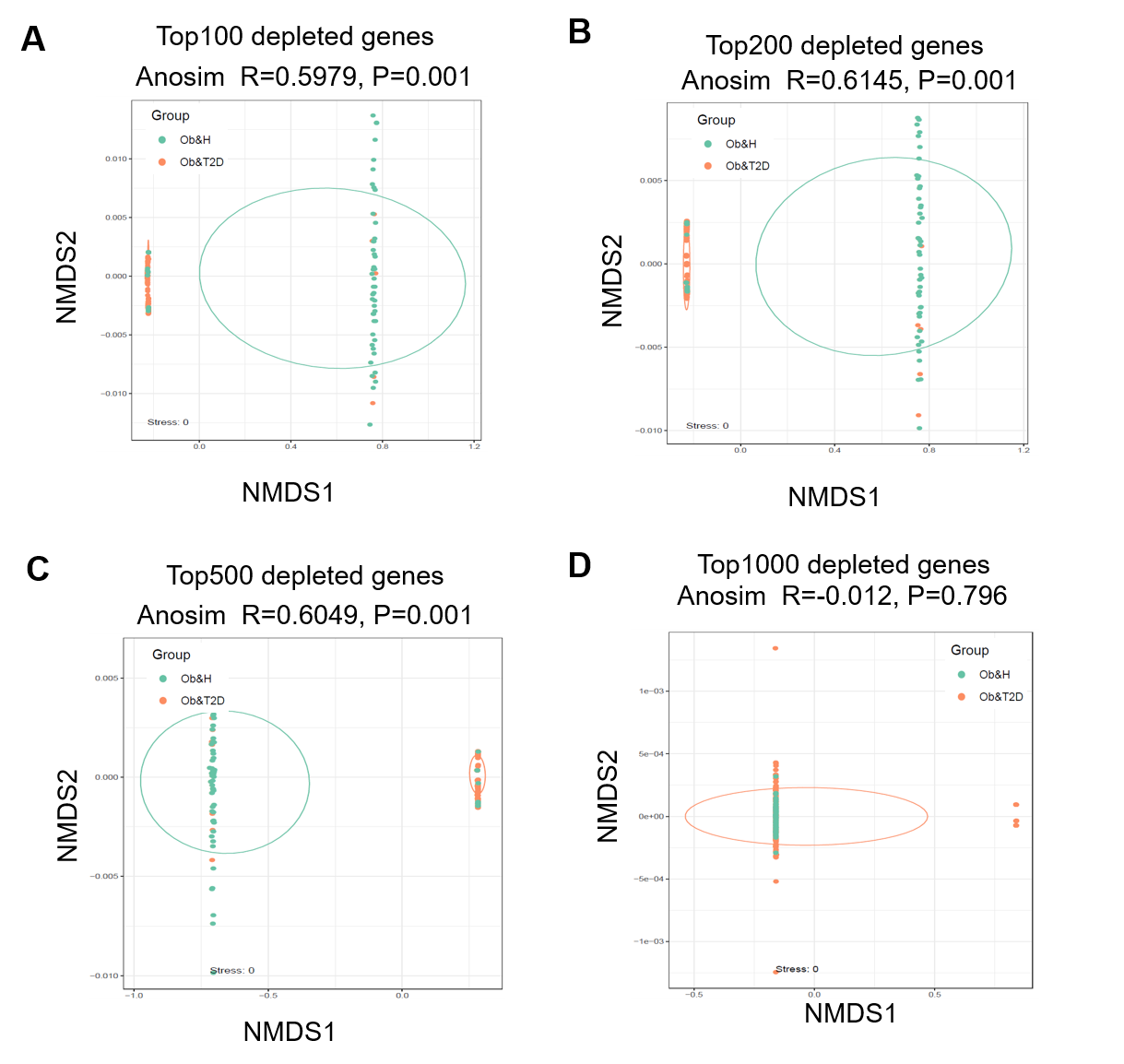

Supplement: Supplementary Figure 6 — NMDS analysis based on the Bray-Curtis distance of top-ranked depleted gene abundance in Ob&T2D. (A–D), NMDS analysis based on the Bray-Curtis distance of top N (N = 100, 200, 500 and 1000) depleted genes abundance in Ob&T2D. Anosim R and P values are shown as indicated. [file Image_6.tif]

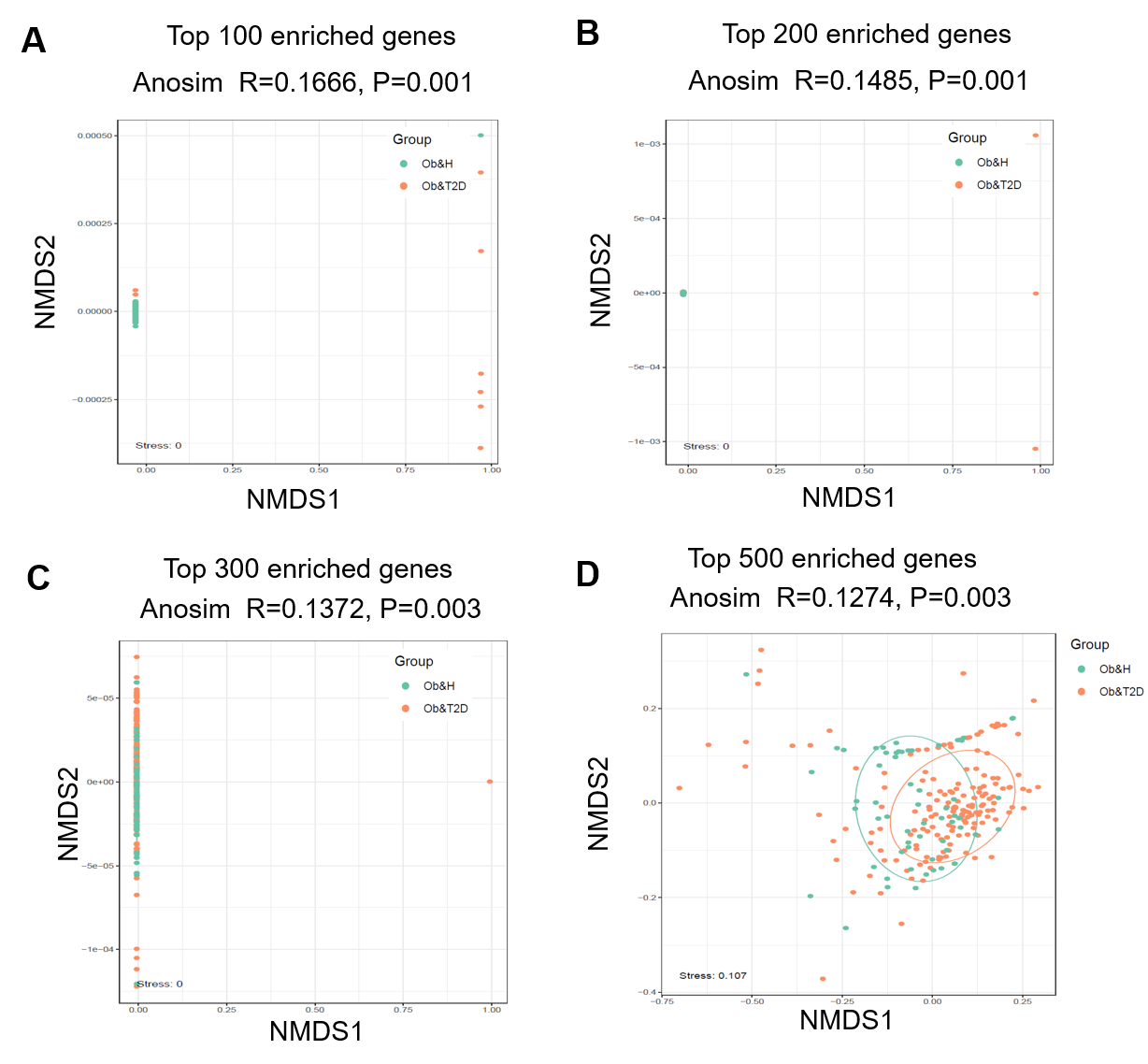

Supplement: Supplementary Figure 7 — NMDS analysis based on the Bray-Curtis distance of top-ranked enriched gene abundance in Ob&T2D. (A–D), NMDS analysis based on the Bray-Curtis distance of top N (N = 100, 200, 300 and 500) enriched genes abundance in Ob&T2D. Anosim R and P values are shown as indicated. [file Image_7.tif]

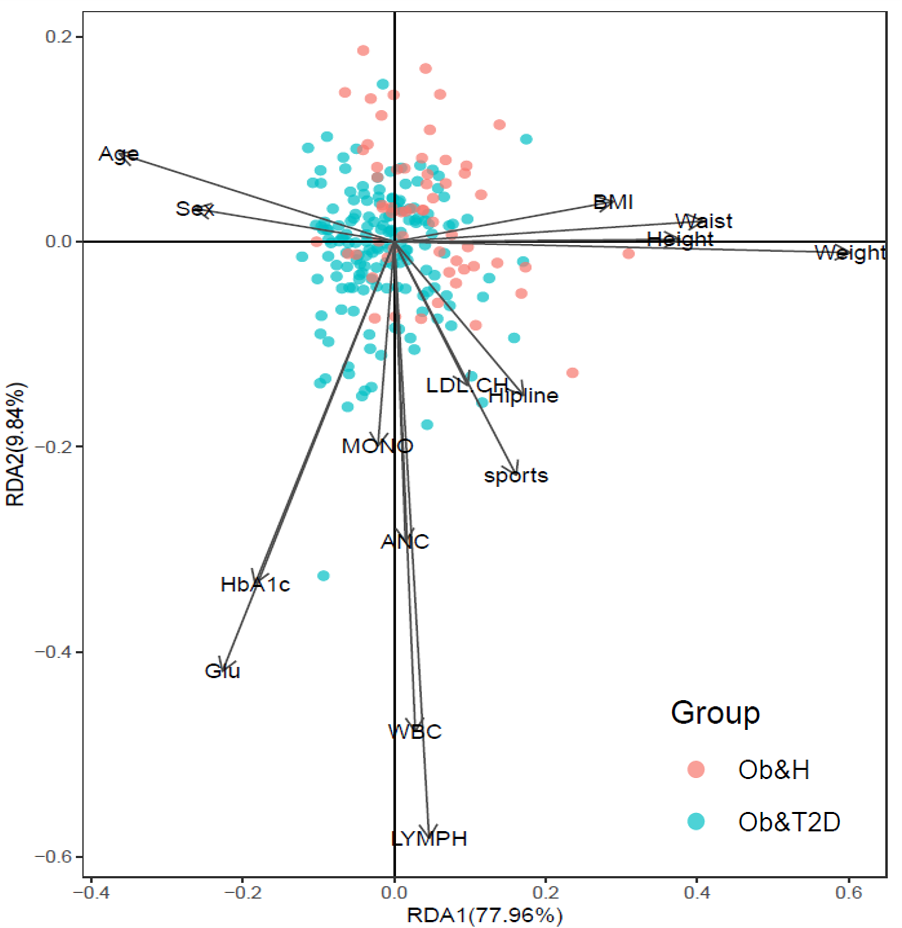

Supplement: Supplementary Figure 8 — Redundancy analysis(RDA) illustrating the relationship between clinal characteristics and microbial gene community. RDA analysis based on the abundance of top 300 depleted genes in Ob&T2D illustrating the relationship between clinical indices and microbial gene community. Glu: glucose level; HbA1c: Hemoglobin A1c; Mono: monocyte; ANC, neutrophils; WBC, white blood cell; LYMPH, ymphocyte. [file Image_8.tif]
